# A Microfluidic Device for Simultaneous Extraction of Plasma, Red Blood Cells, and On-Chip White Blood Cell Trapping

**DOI:** 10.1038/s41598-018-33738-8

**Published:** 2018-10-18

**Authors:** Da-Han Kuan, Chia-Chien Wu, Wei-Yu Su, Nien-Tsu Huang

**Affiliations:** 1 Graduate Institute of Biomedical Electronics and Bioinformatics, National Taiwan University, 10617 Taipei, Taiwan; 2 Department of Electrical Engineering, National Taiwan University, 10617 Taipei, Taiwan

## Abstract

This study reports a microfluidic device for whole blood processing. The device uses the bifurcation law, cross-flow method, and hydrodynamic flow for simultaneous extraction of plasma, red blood cells, and on-chip white blood cell trapping. The results demonstrate successful plasma and red blood cell collection with a minimum dilution factor (0.76x) and low haemolysis effect. The extracted red blood cells can also be applied for blood type tests. Moreover, the device can trap up to ~1,800 white blood cells in 20 minutes. The three components can be collected simultaneously using only 6 μL of whole blood without any sample preparation processes. Based on these features, the microfluidic device enables low-cost, rapid, and efficient whole blood processing functionality that could potentially be applied for blood analysis in resource-limited environments or point-of-care settings.

## Introduction

Blood is composed of plasma, red blood cells (RBCs), white blood cells (WBCs), and platelets, and it contains numerous types of physiological and pathological information about the human body. Currently, complete blood count (CBC) is one of the most common blood tests. The test shows the count of each cell type, cell sizes, the fraction of specific cells in whole blood, and the concentration of various proteins, creatinine, or metabolites. The test gives an overview of patients’ health status^1,2^. For sophisticated CBC analysis, automated haematology analysers and flow cytometers are the most common instruments in hospitals or laboratories. For molecular-level detection, lysed blood cells are drawn into a cuvette for spectrophotometric measurement. Achieving precise CBC measurements usually requires efficient and high-quality processes for the collection and preparation of whole blood samples to avoid any background interference, including centrifugation, fractionation, lysis, or dilution^3^. For example, improper centrifugation could lead to haemolysis, resulting in plasma contamination. Therefore, conventional blood tests are usually time-consuming and require a large volume (~millilitre) of blood, as well as well-trained staff. These requirements limit the access to blood analysis in environments with limited resources or point-of-care settings.

Microfluidics is an ideal technique to simplify whole blood processing based on the capability of integrating different functional elements and potential automation features. Furthermore, due to its miniature channel size, footprint, the required sample volume can be effectively minimised. A comprehensive review of microfluidics for whole blood processing has been shown in previous literatures^3,4^. Basically, microfluidics for blood cells or plasma separation can be categorised into active or passive separation methods^5–7^. Active separation methods exploit external forces (acoustic force^8–10^, dielectrophoretic force^11–13^, magnetic force^14,15^, or combination of above forces^16–19^) to guide targeted cells to a specific direction or position. Passive separation methods simply use differences in cell properties for cell separation, such as the size, shape, or stiffness.

Generally, active separation methods enable high-throughput, high-selectivity cell separation, which is preferred in the sorting or isolation of rare cells (e.g. circulating tumour cell or bacteria) in large amounts of blood samples. However, the samples usually need to be purified cells or RBCs-lysed blood. The reason is the enormous number of RBCs in whole blood, which limits the efficient trapping of WBCs or rare cells. One exception is using a magnetic force to isolate magnetically-labelled WBCs directly from unprocessed whole blood^15^. However, magnetically trapped cells accumulate in a specific region with high magnetic field density, which is not ideal for cell counting or single cell analysis.

In contrast, passive separation methods do not require any cell labelling process or sophisticated micro/nano-fabrication, which makes them more cost-effective and adequate for separating blood cells from plasma, or vice versa. For plasma, one simple microfluidic structure uses a micro-trench along the flow path, which can trap blood cells based on sedimentation and enable plasma purification^20^. Other microchannel geometries for plasma extraction are deterministic lateral displacement (DLD) structures^21^, curved series microchannels^22^, and laminar micro-vortices microfluidics^23^. Another efficient plasma extraction design is using the bifurcation law^24–26^ (or so-called Zweifach-Fung effect) to guide most RBCs into the main microchannel (higher flow rate), and the plasma can be extracted from the side microchannel (lower flow rate)^27^. A similar design uses a slightly wider side microchannel to allow both RBCs and plasma to flow in, while WBCs flow downstream of the main channel^28^. This passive separation mechanism eliminates the complexity of cell labelling. However, the main issue is the lower throughput compared to active separation methods.

Although various microfluidic-based blood cell separation or plasma extraction techniques have been demonstrated, to the best of our knowledge, most methods extract only one kind of blood component by removing or discarding the other components, which limits the detectable parameters in a single whole blood sample. By optimising the bifurcation channel design and adding cellular trapping units, we propose a continuous-flow whole blood processing microfluidic device for isolating multiple blood components. The device contains two types of side channels (with and without packed beads) and a series of hydrodynamic-based WBC trapping units. As a proof of concept, we first measured the absorbance of collected plasma to determine a minimum dilution factor and low haemolysis effect. We then did a blood type test using the extracted RBCs to ensure their characteristics were still well retained^29,30^. Moreover, we analysed the WBC seeding pattern in trapping units to confirm that efficient WBC trapping is possible. Finally, we successfully demonstrated all whole blood processing functions, including extraction of plasma, RBCs, and WBC trapping can be simultaneously performed in a single microfluidic device. The total assay time was just 20 minutes with only 6 μL of whole blood required. To clearly describe the unique feature of our device, we create a table listing important parameters of existing microfluidics for whole blood processing and our work (Table 1). Based on these features, the microfluidic device shows the potential to be applied for blood analysis in resource-limited environments or point-of-care settings.

**Table 1:** A summary of microfluidics for whole blood processing.

Author	Active/Passive	Mechanism	Label-free	Injected Sample	Sample Volume/ Throughpu	Desired target (# of targets)	Performance
Yuchao Chen *et al*.^8^	Active	Acoustic	Yes	Whole blood	10000 μL/min	Platelet (1)	>85% Platelet recovery rate >80% RBC/WBC removal rate
Maria Antfolk *et al*.^9^	Active	Acoustic	Yes	Spiked cancer cells in RBC-lysed and 10X diluted whole blood	100 μL/min	CTC (1)	91.8% MCF7 separation efficiency 84.1% DU145 recovery rate
P. Dow *et al*.^10^	Active	Acoustic	Yes	Spiked bacteria in PBS-diluted whole blood to 20% Hct	10 μL/min	Bacteria (1)	>85% RBC removal rate.45–60% Bacteria yield
Crispin Szydzik *et al*.^11^	Active	Dielectrophoretic	Yes	Whole blood	15 μL in 15 min	Plasma (1)	165 nL undiluted plasma
Anas Alazzam *et al*.^12^	Active	Dielectrophoretic	Yes	Spike cancer cells in sucrose/dextrose medium resuspended whole blood	1.67 μL/min	CTC (1)	95–98% MDA231 separation efficiency
Matthew S. Pommer *et al*.^13^	Active	Dielectrophoretic	Yes	~10X diluted whole blood using LEC buffer	2.5 μL/min	Platelet (1)	95% Platelet purity
Ki-Ho Han *et al*.^14^	Active	Magnetic	Yes	10X diluted whole blood using sodium hydrosulfite	0.083 μL/min	RBC, WBC (2)	93.5% RBC separation efficiency 97.4% WBC separation efficiency.
Macdara T. Glynn *et al*.^15^	Active	Magnetic	No	Spike magnetically-labelled CD4 + cell in whole blood	4 μL in 15 sec	CD4 + cell (1)	93.0% CD4 + cell capture efficiency
Nezihi Murat Karabacak *et al*.^16^	Active/ Passive	Magnetic + Hydrodynamic	No	Add magnetic beads in whole blood.	120 μL/min	CTC (1)	3.8-log depletion of WBC.97% CTC yield.
Hye-Kyoung Seo *et al*.^17^	Active/ Passive	Magnetic + Hydrodynamic	Yes	1000X RBC dilution using PBS	1000 μL/min	WBC, RBC (2)	86.8% RBC separation efficiency 29.1% WBC separation efficiency
Mahdi Mohammadi *et al*.^18^	Active/ Passive	Dielectrophoretic + Hydrodynamic	Yes	Whole blood mix with 1:1 heparin sodium.	2 μL in 7 min	Plasma (1)	100 nL plasma with 99% purity.
C. Wyatt Shield IV *et al*.^19^	Active	Acoustic + Magnetic	No	Spike magnetically-labelled LNCaP cancer cell line in RBC- lysed and PBS resuspended whole blood.	50 μL/min	CTC (1)	89% LNCaP cancer cell line separation efficiency
Ivan K. Dimov *et al*.^20^	Passive	Sedimentation	Yes	Whole blood	5 μL in 10 min	Plasma (1)	99.9–100% blood cell retention.
John A. Davis *et al*.^21^	Passive	Hydrodynamic	Yes	Whole blood	0.4 μL/min	Plasma (1)	100% plasma recovery rate 100% cell removal rate.
Siddhartha Tripathi *et al*.^22^	Passive	Hydrodynamic	Yes	Diluted whole blood using sodium chloride to 7–62% Hct.	500 μL/min	Plasma (1)	99.5% blood cell removal rate.
Elodie Sollier *et al*.^23^	Passive	Hydrodynamic	Yes	20X diluted blood using PBS.	100 μL/min	Plasma (1)	17.8% plasma extraction
Sung Yang *et al*.^27^	Passive	Hydrodynamic	Yes	Whole blood	0.16 μL/min	Plasma (1)	100% plasma purity 15–25% plasma extraction.
Myounggon Kim et al.^28^	Passive	Hydrodynamic	Yes	Whole blood	0.33 μL/min	WBC (1)	96.9% WBC purity 97.2% WBC recovery rate.
This work	Passive	Hydrodynamic	Yes	Whole blood	0.3 μL/min 6 μL in 20 min	Plasma, RBC, WBC (3)	~1.5 μL 0.76-fold dilution, low hemolysed plasma, 1200–1800 trapped WBC

## Results and Discussion

### Device design

**Figure 1:**
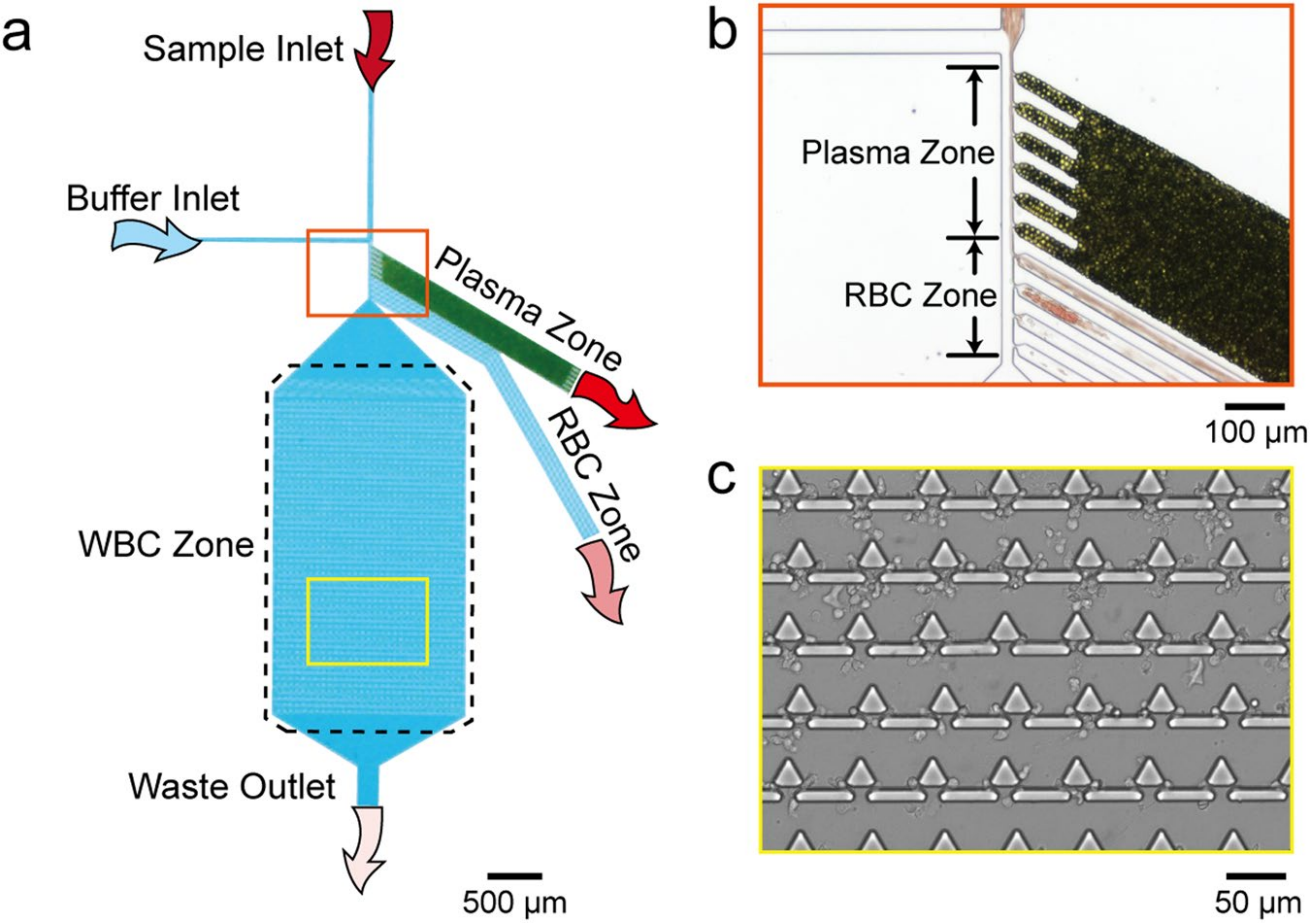
(**a**) Schematic of the microfluidic device, which contains a whole blood sample inlet and a buffer inlet, respectively. After the whole blood sample flowed to the bifurcation region (orange zone), plasma and RBCs were extracted to plasma zone and RBC zone, respectively. WBCs flowed to the main channel and were trapped in the WBC zone. (**b**) Photograph of the plasma zone and RBC zone entrance. Green particles are packed beads, and red particles are RBCs. (**c**) Photograph of the WBC zone with trapped WBCs.

The microfluidic device is composed of a whole blood inlet, buffer inlet, and bifurcation region, which lead to three collection zones for blood components: a plasma zone, RBC zone, and WBC zone. The RBC/WBC separation mechanism is based on the bifurcation law and cross-flow method^28^. Based on a previous study, a flow rate ratio of 1:10 between the whole blood and phosphate buffered saline (PBS) is ideal for RBC/WBC separation. The bifurcation region contains six bead-packed side channels, four-necked side channels, and one main channel for the extraction of plasma, RBCs, and WBC trapping, respectively. To achieve a uniform flow pattern in the bifurcation region, all side channels were tilted at 60 degrees relative to the main channel^31^. A schematic of the microfluidic device is shown in Fig. 1a.

To enable efficient cell separation, the flow pattern of cells needs to be close to the side channel wall of the plasma zone and RBC zone. As shown in Fig. 1b, the whole blood flow pattern is pushed to the side. Typically, RBCs are 6.2~8.2 μm in diameter with 2~2.5 μm in thickness^32^ and WBCs are 7~18 μm in diameter^33^. Due to the densely packed 10-μm beads in the side channel, the effective pore size is 1.55 μm. Therefore, all blood cells would pass by and only plasma can flow into the plasma zone and be collected. Smaller-sized RBCs with higher deformability are then squeezed into the channel of the RBC zone with a 2-μm neck. Finally, WBCs passing by the plasma zone and RBC zone flow across the boundary of two flow patterns and are trapped in the trapping units of the WBC zone (Fig. 1c). The trapping unit is a combination of one triangular pillar and two rectangular pillars with a gap of 2.5 μm. The detailed dimensions of the microfluidic device are listed in Supplementary Figure S1 and Table S1.

### Fluorescent bead simulation

**Figure 2:**
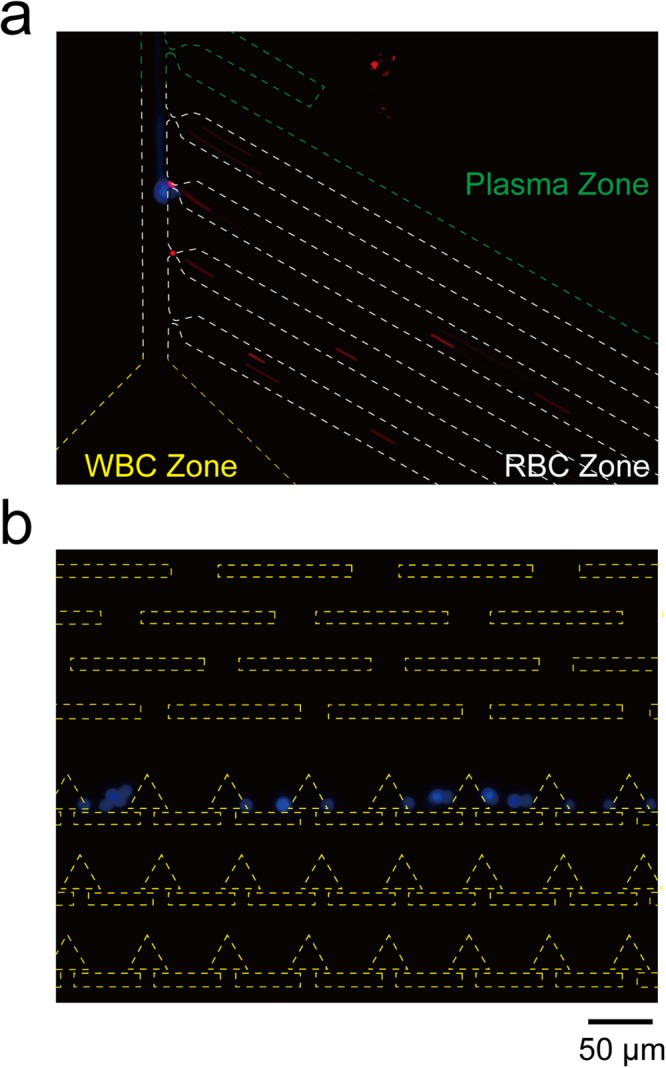
The fluorescence images of beads in the microfluidic device. Green, white and yellow dashed line represent to the plasma zone, RBC zone, and WBC zone, respectively. (**a**) Most 2-μm red fluorescent beads flow into the RBC zone; (**b**) 10-μm blue fluorescent beads flow into the WBC zone.

To verify the RBC/WBC separation functionality of the microfluidic device, we first mixed 1.5 × 10^7^ beads/mL of 2-μm red fluorescent beads and 4.5 × 10^5^ beads/mL of 10-μm blue fluorescent beads to mimic RBCs and WBCs. A fluorescence image of the beads in the bifurcation region and the trapping units of the WBC zone is shown in Fig. 2. The green, white, and yellow dashed lines represent the channel walls of the plasma zone, RBC zone, and WBC zone, respectively. As shown in Fig. 2a, most of the 2-μm beads flowed into the RBC zone due to their small size. Although a few 2-μm beads also flowed into the plasma zone, most of them were stopped at the front end and were not collected in the outlet of the plasma zone.

On the other hand, 10-μm beads passed by the plasma zone and RBC zone and flowed directly into the WBC zone. To smooth the flow pattern, five rectangular pillar rows were placed at the entrance of the WBC zone (Fig. 2b). These pillar rows can also block aggregated cell clusters to prevent any clogging issues in the trapping units. These results show that the bifurcation region design enables successful bead separation, which can be applied for whole blood processing.

### Plasma extraction

We next tested the plasma extraction using four clinical whole blood samples. To ensure that the extracted plasma can be used for biomarker detection, the plasma needs to have (1) a minimum plasma dilution factor and (2) low haemolysis effect. To find out the plasma dilution factor of the device, we first constructed a standard curve of the absorbance versus the dilution factor using a manually diluted plasma sample. First, we extracted plasma by centrifugation at 500 x g for 10 minutes and diluted it with PBS using five different dilution factors (0.2x, 0.4x, 0.6x, 0.8x, 1x).

**Figure 3:**
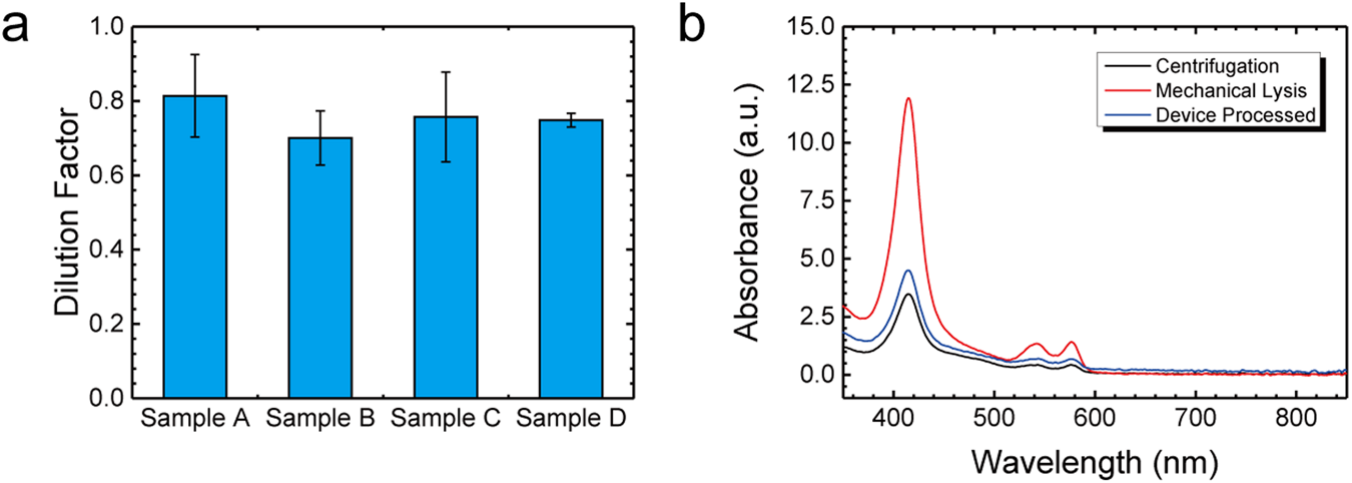
(**a**) Plot of the dilution factor test in 4 clinical samples. Each sample was processed by 3 individual devices. (**b**) The UV-VIS spectra of the plasma extracted from centrifugation (black line), the microfluidic device (blue line), and mechanically lysed whole blood (red line).

We then used a spectrophotometer (Nanodrop One, Thermo Fisher Scientific Inc., MA) to measure the 280-nm absorbance intensity of each dilution factor, which correlates to the total protein concentration due to three amino acids (tryptophan, phenylalanine, and tyrosine)^34^. The protein concentration and composition may vary between different clinical samples, so standard curves of each sample were constructed individually (Supplementary Fig S2). Based on these standard curves, we can then plot the dilution factor of each sample (Fig. 3a). Each sample was processed using three different microfluidic devices. The plasma dilution factors of samples A through D were 0.81x, 0.70x, 0.76x, and 0.75x, respectively. The intra-sample coefficient of variation (CV) was below 16%, indicating a consistent dilution factor in different clinical samples.

To confirm the low haemolysis effect during plasma extraction, we compared the UV-VIS spectra of plasma extracted using a standard centrifugation process (black line), the microfluidic device (blue line), and the mechanically lysed whole blood (red line). The haemolysed plasma sample was collected from mechanically lysed whole blood, followed by vortex and centrifugation at 10,000x g for 10 minutes. As shown in Fig. 3b, the presence of haemoglobin would induce characteristic peaks at 415^35^, 541, and 576 nm^36^. Instead, the spectra of plasma extracted by the standard centrifugation process and the microfluidic device had very similar intensities and profiles. Overall, these results confirm that the plasma extracted by the microfluidic device has a minimum dilution factor and low haemolysis effect and can be used for the detection of various analytes.

### RBC collection for blood type test

**Figure 4:**
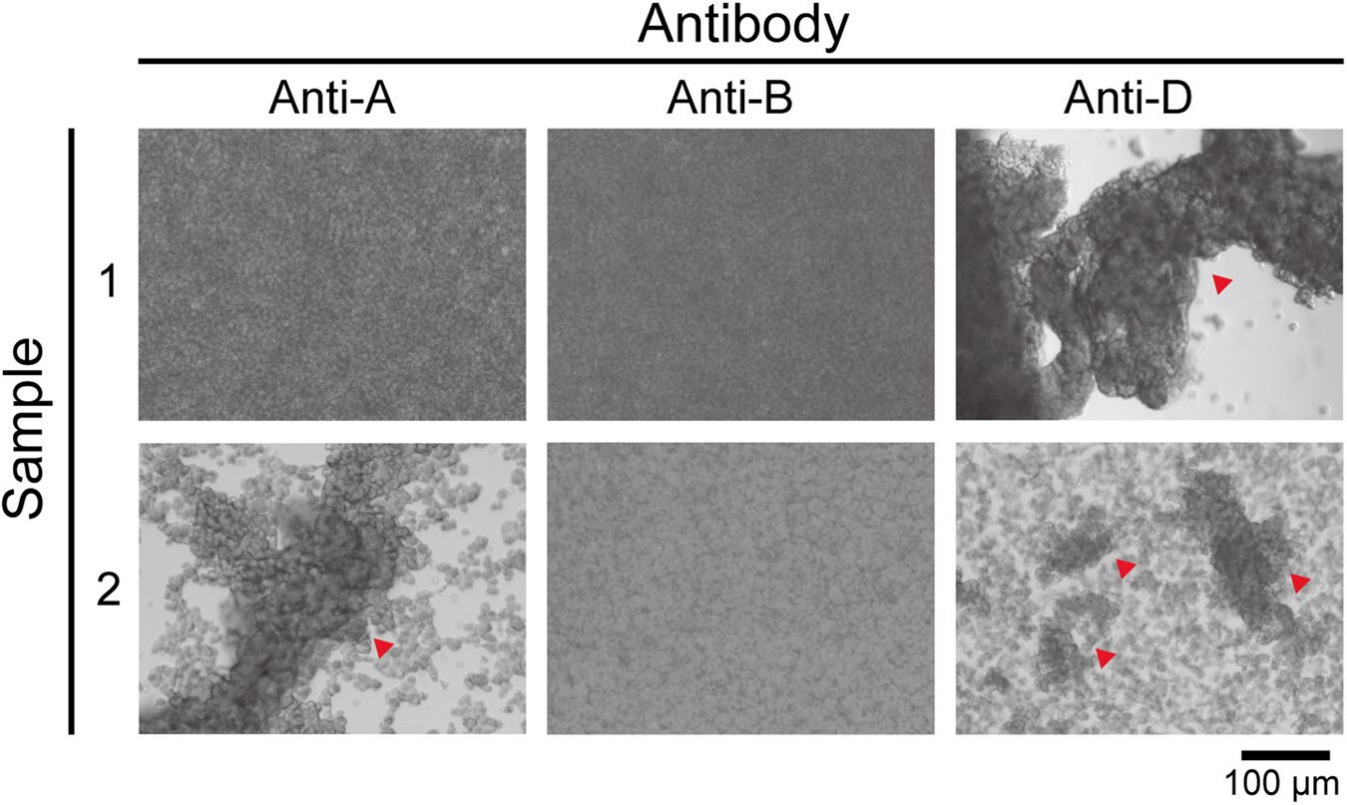
Blood type test images from the extracted RBC solutions of blood sample 1 and sample 2 under anti-A, anti-B, and anti-D treatment. The red triangles indicate blood agglutination.

Next, we performed a blood type test using RBCs extracted from four clinical samples. First, 10 μL of RBC solution extracted from the RBC zone was injected into polydimethylsiloxane (PDMS) chambers treated with anti-A, anti-B, and anti-D antibody solutions. The mixed solutions were incubated for 2 minutes and stirred using pipette tips. As shown in Fig. 4, the RBC solution of sample 1 treated with anti-A and anti-B was still turbid, indicating O-type blood. In sample 2, the RBC agglutination (red triangle) can be easily observed in the anti-A-treated RBC solution, but it remained turbid in anti-B treatment, indicating A-type blood. When both sample 1 and 2 were treated with anti-D, the RBC agglutination result indicated that both samples are Rh-positive blood. We used the same method to identify the blood types of sample 3 and 4, and both samples were O-type and Rh-positive blood. A summary is presented in Supplementary Table S2.

### White blood cell trapping

Then, we tested the WBC trapping performance of the microfluidic device. To evaluate the cell seeding pattern, we divided the WBC zone into top, middle, and bottom regions. Each region contains 464 trapping units in the 1-mm x 1.7-mm area. To use all trapping units efficiently and prevent any potential cell clogging issues, we designed a 30-μm gap at the end of each row. Therefore, when trapping units of one row were fully occupied, the following cells could flow through the gap to the next row. Based on this design, we observed that the WBCs were first captured at the top region and gradually successively moved toward the middle and bottom regions.

**Figure 5:**
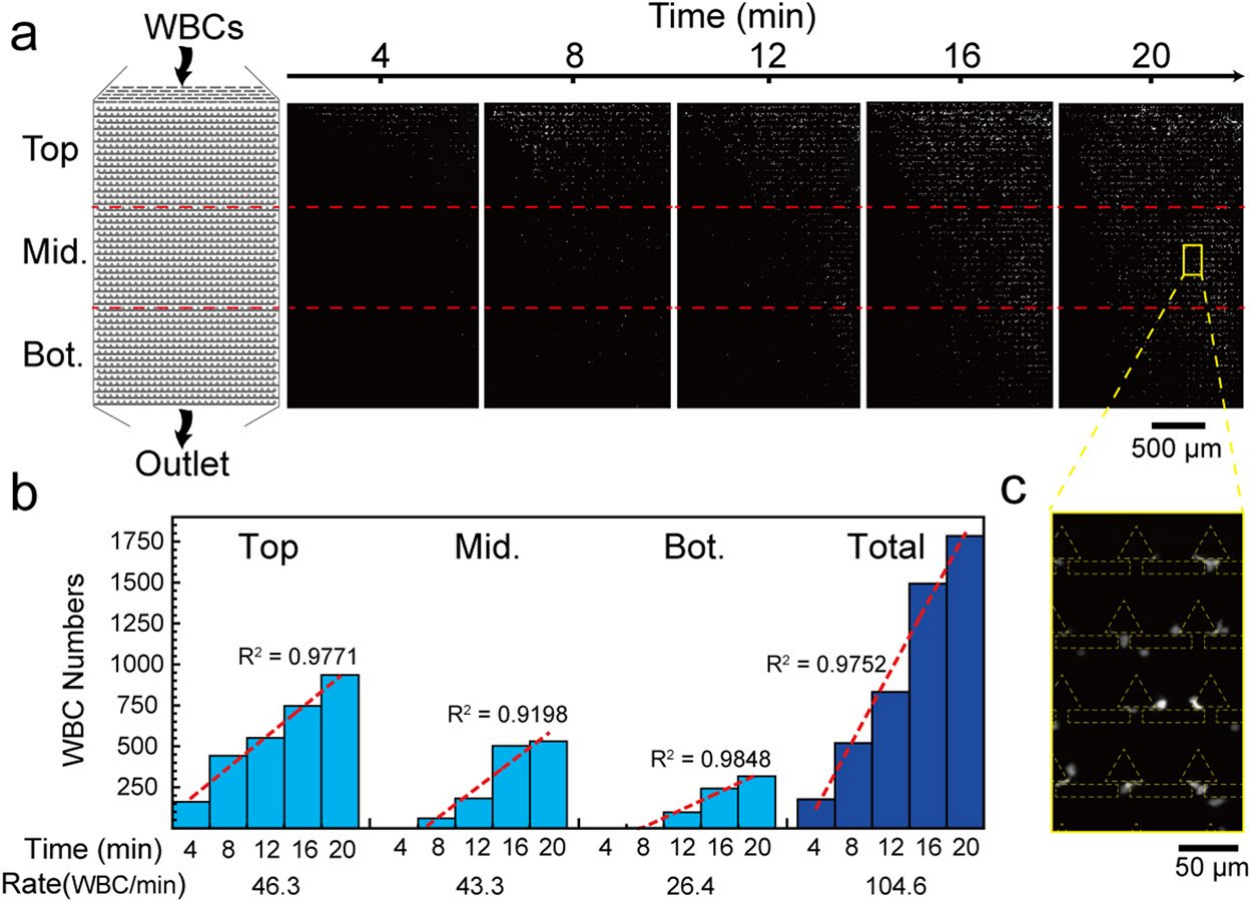
WBC seeding pattern in the trapping units of the microfluidic device. (**a**) Schematic and time-lapse fluorescence images of WBC zone. (**b**) Trapped WBC numbers in top, middle, and bottom regions at different time points. The red dashed lines are the regression lines of each region, representing the WBC capture rate. (**c**) Enlarged image of the yellow box showing the WBC seeding profile.

Figure 5a shows time-lapse fluorescence images of the WBC zone at 4, 8, 12, 16, and 20 minutes. The images were analysed by Image J software and used to determine the numbers of WBCs in the top, middle, bottom, and overall regions of the WBC zone. As shown in Fig. 5b, the WBCs start to be captured in the top, middle, and bottom regions at t = 0, 6.61, and 7.61 minutes, and the capture rates were 48.3, 43.3, and 26.3 WBCs/min, respectively. There is a similar capture rate in the top and middle regions, indicating that the trapping efficiency would not be affected by preceding cell-occupied trapping units.

In contrast, the capture rate in the bottom region was 0.6-times lower than in the top and middle regions. We suspect that the reason is that the trapping units of the top and middle regions were not fully occupied yet. The number of trapped cells in the whole device shows an almost linear curve, indicating a uniform cell seeding pattern. For the whole region, 1,784 WBCs were captured from 6 μL of whole blood with a total capture rate of 104.6 WBCs/min.

### Simultaneous extraction of plasma, RBC and WBC trapping in a single microfluidic device

**Figure 6:**
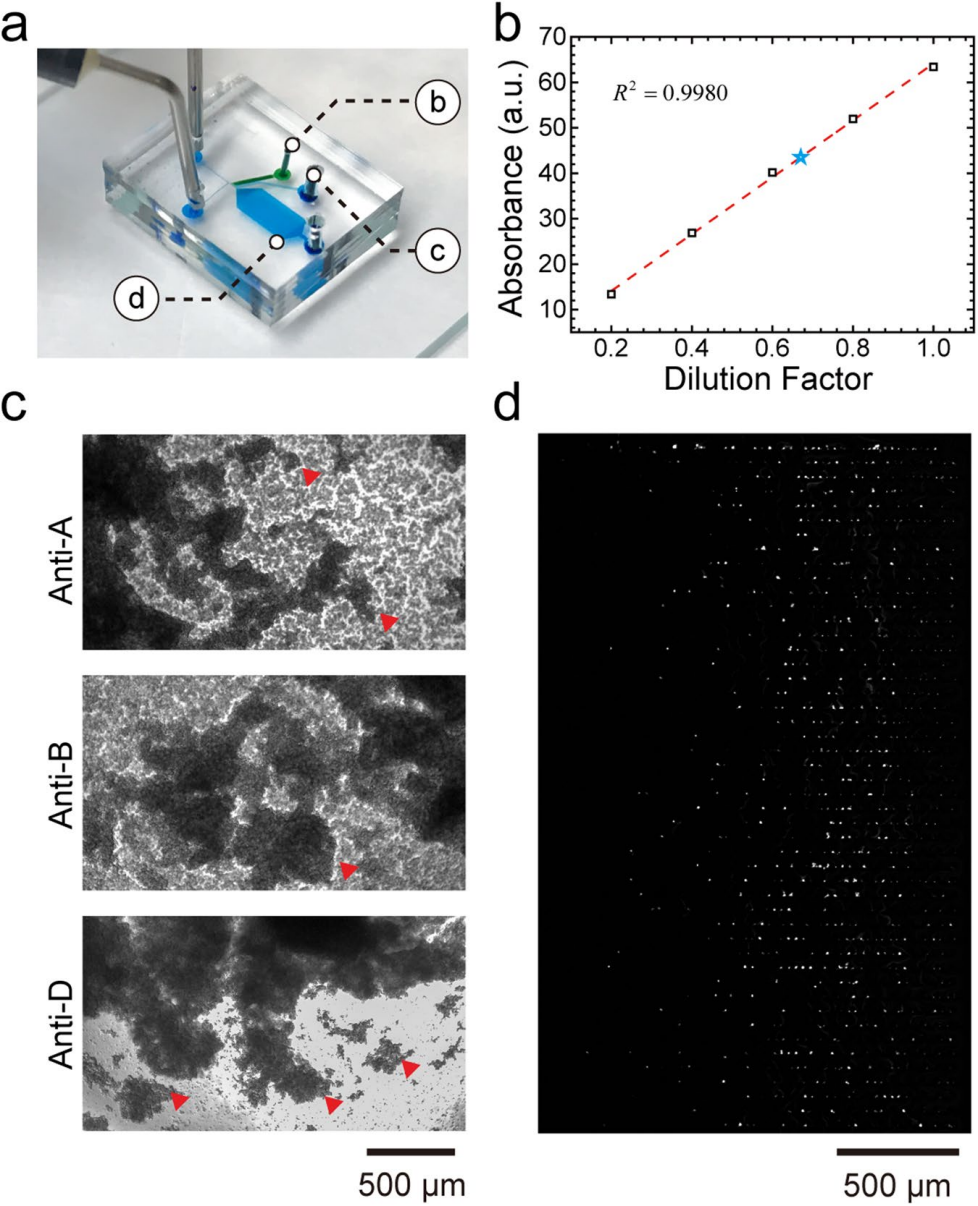
Simultaneous extraction of plasma, RBCs and on-chip white blood cell trapping. (**a**) Image of the microfluidic device showing the position of each zone. (**b**) Standard curve of the absorbance intensity versus dilution factor of manually diluted blood (black rectangles and red dashed line). The blue star represents the absorbance and corresponding dilution factor (0.67x) of extracted plasma in this test. (**c**) Blood type test images from the extracted RBC solution. The red triangles represent blood agglutination. (**d**) WBC seeding pattern in the WBC zone.

Finally, we aim to demonstrate the microfluidic device enables the simultaneous extraction of plasma, RBC and on-chip WBC trapping. Same as previous protocols, 6 μL whole blood stained with Calcein AM was loaded into the microfluidic device in 20 minutes. Plasma and RBCs were then extracted from the plasma zone and RBC zone, respectively. In the meantime, WBCs were continuously trapped in the WBC zone. The image of microfluidic device consisted of each zone is shown in Fig. 6a. Once the experiment was done, we then characterized the plasma dilution factor as 0.67x (Fig. 6b) and blood type as AB-type Rh-positive (RBC agglutination in all anti-A, anti-B, and anti-D treatment) (Fig. 6c). As shown in Fig. 6d, WBCs were uniformly seeded in the WBC zone and the total number of trapped WBCs was 1,221, which is similar to the previous test. In summary, all three whole blood processing functions can be performed simultaneously without any interference in a single microfluidic device.

## Conclusion

We have developed a microfluidic device that enables the simultaneous extraction of plasma and RBCs as well as on-chip WBC trapping. The results demonstrated two important features. First, the bifurcation region contains two types of side channels that can extract plasma and RBCs separately. The extracted plasma has a minimum dilution factor (0.755x) and low haemolysis effect. Furthermore, the extracted RBCs can be applied for blood type tests. Second, we added a series of hydrodynamic-based WBC trapping units downstream of the main channel. Based on the carefully designed geometry of the trapping units, the device can trap up to ~1,800 WBCs in 20 minutes. The trapped WBCs could potentially be used for various cellular analyses, such as drug screening, DNA extraction, or cell viability tests. Compared to other existing microfluidic method for whole blood processing, our microfluidic device can directly process extremely low-volume (6 μL) whole blood without any pretreatment (e.g. dilution or lysing), pre-labeling (e.g. fluorophores or magnetic beads conjugation) requirement or external fields (e.g. optical, electrical or magnetic fields) to assist plasma/cell separation. In the future, we aim to further increase the collection throughput of the three blood components. Furthermore, we plan to integrate an automated microfluidic flow control system that has already been developed in our laboratory, which would precisely control all flow conditions and make the system more user-friendly^37^. Overall, we envision that this device can become a low-cost, rapid, and multi-functional tool for whole blood processing in resource-limited environments or point-of-care settings.

## Methods and Materials

### Device fabrication

The microfluidic device was made of PDMS and fabricated using a standard soft lithography process. Briefly, PDMS prepolymer (Sylgard-184, Dow Corning) composed of precursor A, precursor B, and silicon oil at a ratio of 10:1:0.3 was poured onto a silicon mould with channel structures fabricated by the photolithography process. After heating in an oven at 60 °C for 4 hours, the fully cured PDMS structure was peeled off the mould. The inlet and outlet of the microchannel were then punched with 0.5-mm and 1-mm biopsy punches, respectively, followed by bonding to a glass slide using oxygen plasma (Plasma Cleaner PDC-001, Harrick Plasma) at 45 W for 60 s.

After bonding, the first six side channels were packed with 10-μm polystyrene beads (18337-2, Polysciences Inc., PA) from the outlet using a pneumatic pump (SH-P100, Shishin, Taiwan). To enable smooth and uniform bead packing, the bead mixture was first diluted with deionised (DI) water to 1.5 × 10^6^ beads/mL and loaded into channels at two injection speeds (52 and 520 μL/min). The first injection allows gradual bead accumulation at the necked structure. The second injection is used to further squeeze the packed beads and increase their packing density. After the bead packing process, the device was placed into the oven at 60 °C for another 8 hours to remove all residual solvents. Before usage, the device was immersed in DI water with 3% Pluronic solution for 15 minutes to prevent the formation of any air bubbles and non-specific adhesion of blood cells.

### Sample preparation and blood collection

In the simulation experiment using fluorescent beads, we used 2-μm red fluorescent beads (19508-2, Fluoresbrite® Polychromatic Red Microspheres, Polysciences Inc., PA) and 10-μm blue fluorescent beads (F8829, FluoSpheres™ Polystyrene Microspheres, ThermoFisher Scientific, MA). The clinical samples of whole blood were collected using a blood collection tube coated with ethylenediaminetetraacetic acid (EDTA) (BD Vacutainer® spray-coated with K2EDTA, BD, NJ). The study was approved by the Ethical Committee of National Taiwan University Hospital (201505009DINC), and all subjects were given written informed consent. All clinical experiments were performed in accordance with the relevant guidelines and regulations. The experiments were finished within 24 hours after sample collection. To visualise and count trapped WBCs in the device, the whole blood sample was stained by the cell viability assay dye Calcein AM. Calcein AM was diluted with diethyl sulfoxide (DMSO) and mixed with the whole blood to a final concentration of 10 μM. The whole blood sample was then incubated for 30 minutes at 37 °C for the following experiments.

For the blood type test, we injected 20 μL of anti-A, anti-B, and anti-D antibody solution (TECO Diagnostics, CA) into three 5-mm-diameter PDMS chambers. For A-type blood, antigen-A on the RBC surfaces interacts with the anti-A antibody, resulting in RBC agglutination. Similarly, agglutination in anti-B-treated solution represents B-type blood. Agglutination in both anti-A and anti-B treated solution indicates AB-type blood. However, if no agglutination occurs at all, the blood is type O. For the Rh blood group system, blood type is identified by the anti-D antibody.

### Whole blood processing protocol using the microfluidic device

A 6 μL amount of whole blood sample and 60 μL of PBS solution were injected into the microfluidic device using two syringe pumps (Fusion 200, Chemyx Inc., TX) via the whole blood inlet and buffer inlet, respectively. The flow rates of whole blood and PBS were first set at 0.3 and 1 μL/min, respectively. In the first 3 minutes, a stable boundary layer between the whole blood and PBS was formed, and the flow pattern in the main channel became stable. The flow rate of PBS was then gradually increased to 3 μL/min to reduce the width of the whole blood flow. To collect plasma and RBCs, a pipette tip was inserted in the outlets of both side channels. Once the whole assay process was finished, the pipette tips can be directly unplugged for different tests. The WBC trapping process was recorded by a charge-coupled device (CCD) camera (DP-80, Olympus) under an inverted fluorescence microscope (IX73, Olympus, Japan).

## Electronic supplementary material

Supplementary information
