# Supplementary material for: A Microfluidic Device for Simultaneous Extraction of Plasma, Red Blood Cells, and On-Chip White Blood Cell Trapping
Source: Sci Rep. 2018 Oct 18;8:15345. doi: 10.1038/s41598-018-33738-8 (PMC6194116; doi:10.1038/s41598-018-33738-8)
Supplement: Supplementary file 1 — Supplementary information [file 41598_2018_33738_MOESM1_ESM.docx]

Supplemental Information

A Microfluidic Device for simultaneous Extraction of Plasma, Red Blood Cells and on-chip White Blood Cells Trapping

Da-Han Kuan ^1^, Chia-Chien Wu ^1^, Wei-Yu Su ^1^ and Nien-Tsu Huang^1,2*^

^1^ Graduation Institute of Biomedical Electronics and Bioinformatics, ^2^ Department of Electrical Engineering, National Taiwan University, 10617 Taipei, Taiwan


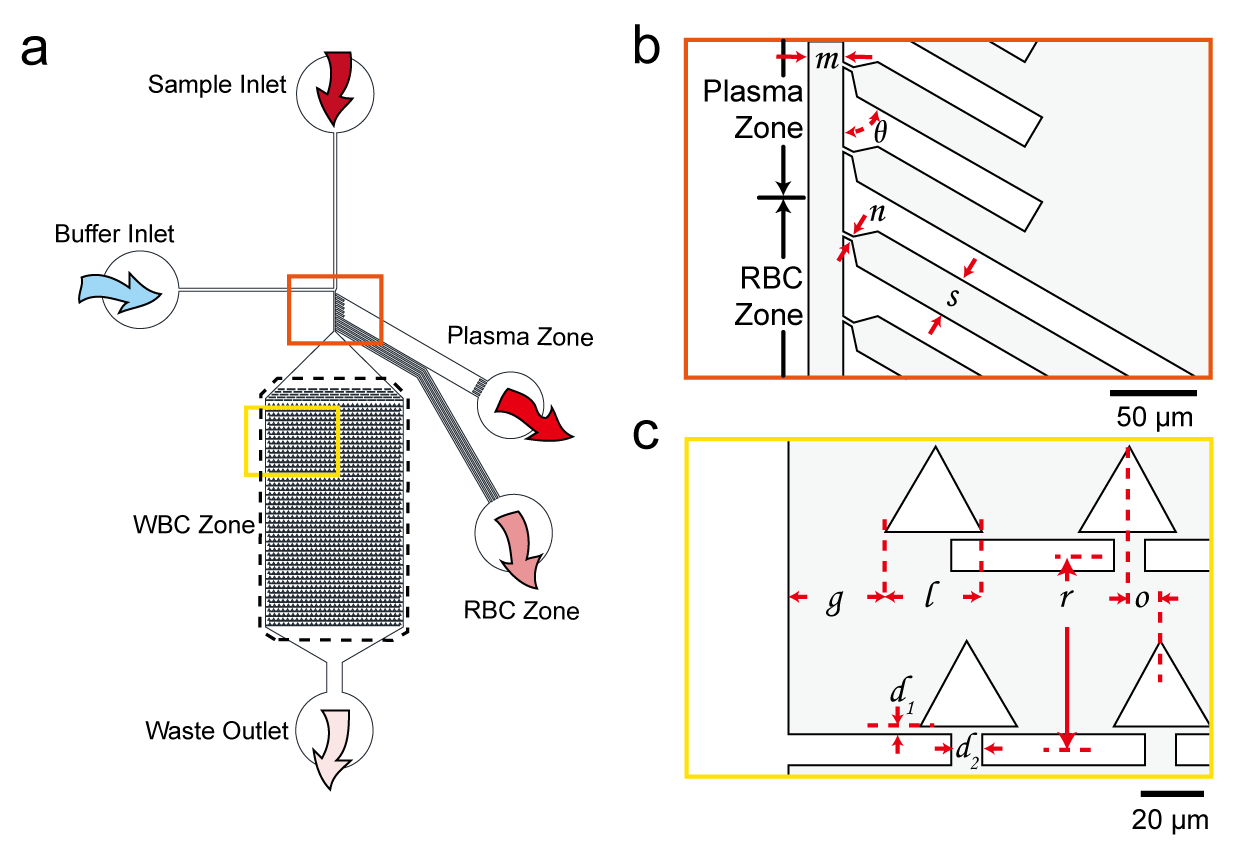


Figure S1. The detailed dimension of microfluidic device. The corresponding values are summarized in Table S1.

Table S1. The definition and values of the symbols in Figure S1

| Symbol | Definition | Value (μm) |
| --- | --- | --- |
| m | Main channel width | 20 |
| θ | Side channel tilt angle | 60^o^ |
| n | Neck channel width | 3 |
| s | Side channel width | 23.5 |
| d_1_ | Triangle-Rectangle silt distance | 2.5 |
| d_2_ | Rectangle-Rectangle silt distance | 10 |
| l | Edge length of rectangle | 30 |
| r | Row-to-row distance | 60 |
| o | Offset distance | 10 |
| g | Gap width | 30 |

Table S2. The blood type test results of 4 clinical samples. + stands for clump images and – stands for turbid images

| Sample | Antibody | | | Blood Type |
| --- | --- | --- | --- | --- |
|  | **Anti-A** | **Anti-B** | **Anti-D** |  |
| 1 | **－** | **－** | **＋** | O Rh^+^ |
| 2 | **＋** | **－** | **＋** | A Rh^+^ |
| 3 | **－** | **－** | **＋** | O Rh^+^ |
| 4 | **－** | **－** | **＋** | O Rh^+^ |
| 5* | **＋** | **＋** | **＋** | AB Rh^+^ |

*Performed in the simultaneous whole blood processing test


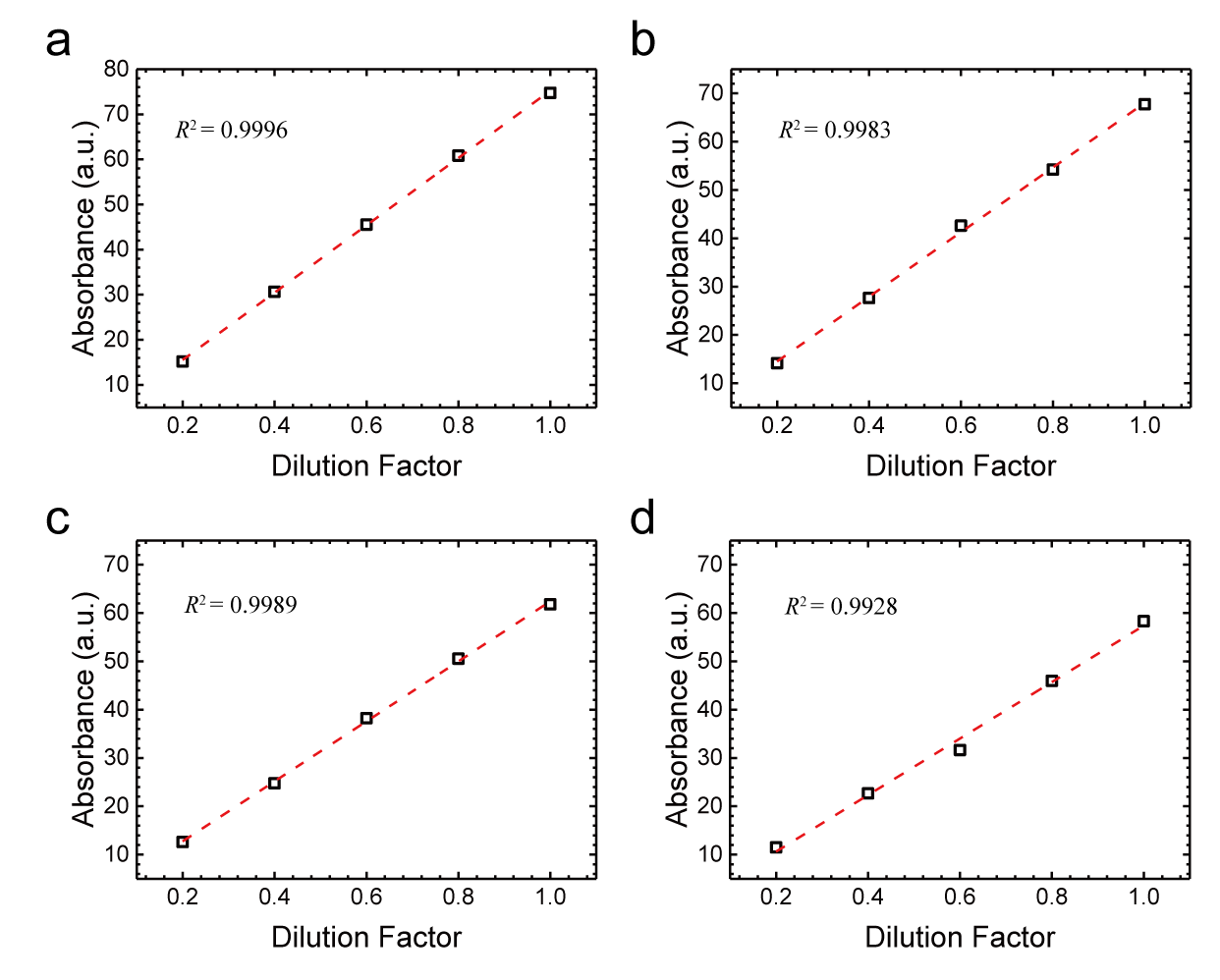


Figure S2. The standard curves of the absorbance intensity versus the dilution factor of manually diluted blood from 4 clinical samples.
